# Long non-coding RNA DUXAP8 promotes the cell proliferation, migration, and invasion of papillary thyroid carcinoma via miR-223-3p mediated regulation of CXCR4

**DOI:** 10.1080/21655979.2021.1882134

**Published:** 2021-02-15

**Authors:** Yan Liu, Hejia Zhang, Hui Wang, Jiarui Du, Peng Dong, Meihan Liu, Yuanqiang Lin

**Affiliations:** Department of Ultrasound, China-Japan Union Hospital of Jilin University, Changchun, People’s Republic of China

**Keywords:** Papillary thyroid carcinoma, long non-coding RNA DUXAP8, miR-223-3p, CXCR4, proliferation, migration and invasion

## Abstract

Papillary thyroid carcinoma (PTC) is a differentiated type of thyroid malignancy with a high incidence. Long non-coding RNA (lncRNA) DUXAP8 has been reported to participate in the proliferation, migration, and invasion of several cancer types. However, its association with PTC has not yet been reported. The current study aimed to investigate the role of DUXAP8 in PTC and revealed the underlying mechanisms. The expression of DUXAP8 was knocked down in two PTC cell lines and the effects of DUXAP8 on the PTC biological behavior were examined by cell counting kit-8 (CCK-8), wound healing, and transwell invasion assays. Luciferase reporter assay was used to detect the binding activity between miR-223-3p and DUXAP8. We found that knockdown of DUXAP8 inhibited the proliferation, migration, and invasion of PTC cells. DUXAP8 could sponge miR-223-3p through the specific binding site. CXCR4 was a target of miR-223-3p. The malignant phenotypes of the PTC cells were suppressed by the over-expression of miR-223-3p. Moreover, miR-223-3p inhibition or CXCR4 over-expression partly restored the proliferation, migration, and invasion activities of DUXAP8-downregulated PTC cells. The results evidenced that DUXAP8 acted as an oncogene in PTC, these effects seemed to partly dependent on the miR-223-3p/CXCR4 axis.

## Graphical abstract

**Figure:**
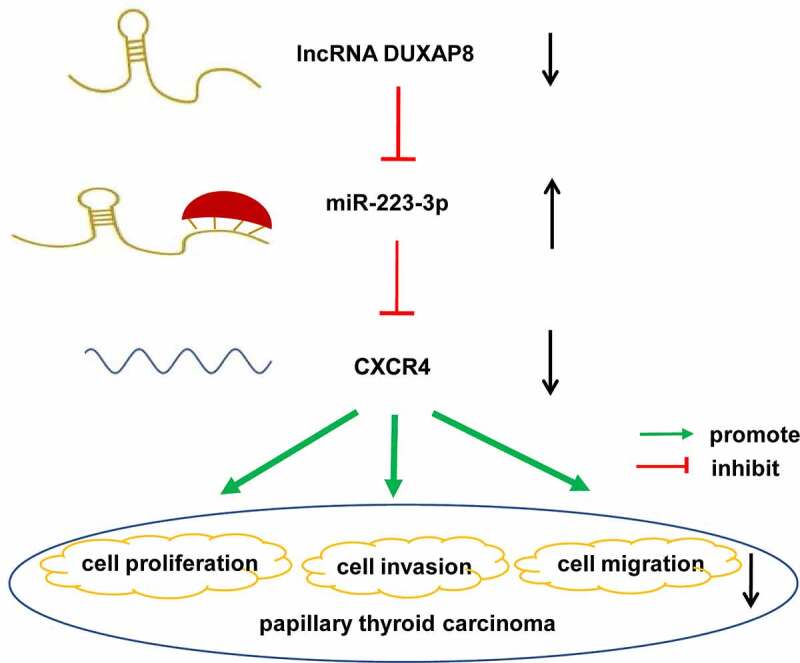

## 1. Introduction

Papillary thyroid carcinoma (PTC), a differentiated type of thyroid malignancy whose incidence has tripled over the past three decades [1], accounts for more than 80% of thyroid cancer [2,3]. Large-scale genomic characterization of PTC has revealed that genetic alterations play a critical role in the tumorigenesis of this disease [4]. A study has shown that the mitogen-activated protein kinase (MAPK) signaling pathway activated by somatic mutations and gene fusions was regarded as the primary molecular aberrations for PTC [5]. However, the carcinogenesis of PTC is a complex biological process marked by several molecular abnormalities, and the reason for its high prevalence remains poorly understood [6]. Moreover, some patients with differentiated thyroid cancer onset still have distant metastases, followed by a reduced survival rate to less than 50%[7]. Hence, there remains a compelling need to explore novel molecular targets to understand the pathogenesis and progression of PTC.

Long non-coding RNA (lncRNA), more than 200 nucleotides in length, can participate in the regulation of various biological processes in cells [8]. Since Salmena et al. put forward the hypothesis of competitive endogenous RNA (ceRNA) in 2011, people have gained a new understanding of miRNA and lncRNA in the mechanism of regulation, which might become a means to explain the course of diseases and bring opportunities for new treatments [9]. For example, Zhang et al. demonstrated that targeted inhibition of lncRNA H19 could block anaplastic thyroid carcinoma growth and metastasis [10]. Ding et al. indicated that high lncRNA 00511 expression level may be correlated with poor prognosis and high incidence of metastasis [11]. LncRNA DUXAP8 is about 2107 bp in length. In the past two years, studies have reported that lncRNA DUXAP8 promoted the proliferation and invasion of renal cell carcinoma [12], esophageal squamous cell carcinoma [13], gastric cancer [14], non-small cell lung cancer [15], and other tumors. Also, we found through public database analysis that the high expression of lncRNA DUXAP8 was related to the poor prognosis of thyroid cancer. However, the molecular mechanism of lncRNA DUXAP8 in regulating PTC has not yet been reported.

Previous studies have demonstrated that lncRNA regulated the gene expression via the regulation of target genes by lncRNA or the bind with certain RNA-binding proteins [15,16]. For instance, a report showed that miR-126 was down-regulated by lncRNA DUXAP8 to enhance renal cell carcinoma progression [12]. Liu et al. indicated that silencing lncRNA DUXA8 inhibited the progression of lung adenocarcinoma via targeting miR-26b-5p [17]. Research in non-small-cell lung cancer indicated that lncRNA DUXAP8, linked with miR-409-3p, exerted the role of promoting cell migration and glycolysis through up-regulating the expression of HK2 and LDHA [18]. Another study showed in hepatocellular carcinoma that lncRNA DUXAP8 sponged miR-490-5p to induce the expression of BUB1, which promoted cell proliferation and migration [19]. Hence, we further want to find out the target gene which is regulated by lncRNA DUXAP8 in PTC.

It is worth noting that miR-223-3p can inhibit the proliferation and invasion of osteosarcoma [20], breast cancer [21], glioma [22], and other malignant tumors, and its role in PTC has also not been reported. Moreover, the previous research in our group proved that CXC chemokine receptor 4 (CXCR4) can mediate the CXCL12 signal to promote PTC cell migration, invasion, and EMT [23]. Through searching for ENCORI, we found that both CXCR4 and lncRNA DUXAP8 have miR-223-3p binding sites. Hence, we hypothesized that lncRNA DUXAP8 might attenuate PTC via miR-223-3p mediated regulation of CXCR4. To verify this hypothesis, we conducted a DUXAP8-silenced model in two PTC cell lines to investigate the role of DUXAP8 in PTC and reveal the underlying mechanisms.

## 2. Materials and methods

### 2.1. Cell lines culture

The TPC-1 cells purchased from Procell (Wuhan, China) were cultured in RPMI-1640 medium (31800–014, Gibco, USA) supplemented with 10% fetal bovine serum (FBS, Biological Industries, Israel). The IHH-4 cells purchased from Cobioer (Nanjing, China) were cultured in Dulbecco’s modified eagle medium (DMEM, Gibco, USA)/RPMI-1640 medium supplemented with 10% FBS. The GLAG-66 cells purchased from Cellcook (Guangzhou, China) were cultured in DMEM containing 10% FBS. The 293 T cells were purchased from Zhong Qiao Xin Zhou Biotechnology (Shanghai, China). These cells were all maintained in humidified air at 37°C with 5% CO_2_.

### 2.2. Cells transfection

DUXAP8 specific small interfering RNA (siDUXAP8), siRNA negative control (siNC), NC mimic, miR-223-3p mimic, and miR-223-3p inhibitor, purchased from JTS scientific (Wuhan, China) were stored at −20°C. GLAG-66 and TPC-1 cells were seeded in 6-well plates and incubated overnight. Cell transfection was performed according to the instructions of the Lipofectamine 2000 reagent (Invitrogen, California, USA). The interference sequences of siDUXAP8 were listed in Table 1.

**Table 1.** The interference sequences of siDUXAP8

Name	Sequences	
siDUXAP8#1	CAGCATACTTCAAATTCACAGCAAA	
siDUXAP8 #2	AAGATAAAGGTGGTTTCCACAAGAA	

### 2.3. Quantitative realtime-polymerase chain reaction (qRT-PCR)

Total RNA was extracted using RNA pure Total RNA Kit9 (RP5612, BioTeke, China). cDNA synthesis was performed using SuperScript M-MLV reverse transcriptase (2641A, Takara, Dalian, China) with Exicycler^TM^ 96 Real-Time Quantitative Thermal Block (BIONEER, Korean). qRT-PCR was performed in 20 μl reactions on cDNA with SYBR Green PCR reagents (Solarbio, China) and Taq HS Perfect kits (TaKaRa, Dalian, China) using Exicycler^TM^ 96 Real-Time Quantitative Thermal Block. The thermal cycler conditions were 40 cycles of 94°C for 2 minutes, 94°C for 10 seconds, 60°C for 15 seconds, and 72°C for 15 seconds. The level of target mRNA was normalized to the level of β-actin. U6 was used as an endogenous control for miRNA. We used a 2^−∆∆CT^ method to assay the relative expression levels. The primer sequences were listed in Table 2.

**Table 2.** The sequences of primer

Name	Sequences	
lncRNA DUXAP8 F	CACCACAGTTACTTTATCCCTT	
lncRNA DUXAP8 R	CCTTTAGACCCATTCTCGTAT	
CXCR4 F	CACGCCACCAACAGTCAGA	
CXCR4 R	CACAACCACCCACAAGTCA	
hsa-miR-223-3p F	TGTCAGTTTGTCAAATACCCC	
hsa-miR-223-3p R	GCAGGGTCCGAGGTATTC	
β-actin F	CTTAGTTGCGTTACACCCTTTCTTG	
β-actin R	CTGTCACCTTCACCGTTCCAGTTT	
U6 F U6 R	GCTTCGGCAGCACATATACT GTGCAGGGTCCGAGGTATTC	

### 2.4. Cell counting kit-8 (CCK-8) assay

GLAG-66 and TPC-1 cells were seeded in the 96-well plate at the density of 3 × 10^3^cells/well. After cell transfection, the cells were cultured for 0 h, 24 h, 48 h, 72 h, followed by incubation with 10 μl CCK-8 solution (KeyGENBioTECH, Nanjing, China) at 37 °C or 1 h. Then the OD value at 450 nm was measured by ELX-800 Absorbance Microplate Reader (BioTek, Winooski, VT, USA).

### 2.5. Wound healing assay

Cells were cultured in an incubator at 37 °C and 5% CO_2._ When the cell density reached about 90%, the medium was replaced with serum-free medium, and 1 μg/ml mitomycin C (Sigma-Aldrich, USA). After cultured for 1 h, we used a 200 μl pipette tip to scratch the cells and cultured the cell with a serum-free medium for an additional 24 h. Then, we observed the wounded gaps under a 100× microscope, and took pictures in groups at 0 h and 24 h, calculated the wound healing ratio of each group.

### 2.6. Transwell invasion assay

Transwell chambers, purchased from Corning (USA), were used for the invasion experiments. The matrigel glue (BD, USA) was diluted with the serum-free medium in a ratio of 1:3 on ice. Took out the transwell cell and placed it in a 24-well plate, coated it on the cell membrane with 40 μl of pre-diluted matrigel gel, and placed it in a 37 °C incubator 2 h to set the glue. For invasion experiments, 200 μl of cell suspension (2 × 10^4^ cells for each well) were seeded into upper chambers. 800 μl of medium with 10% FBS (Beyotime, China) was added to the lower chamber to be a chemoattractant. Then we placed the 24-well plate in a cell incubator at 37 °C, 5% CO_2,_ and saturated humidity. After incubation for 48 h, the transwell cell was washed twice with PBS, fixed at room temperature (RT) with 4% paraformaldehyde for 25 min, stained with 0.4% crystal violet staining (Amresco, USA) solution for 5 min, and rinsed with distilled water. At last, the cells invaded into the lower layer of the microporous membrane were counted and scored under a microscope.

### 2.7. Luciferase reporter assay

The sequences of DUXAP8 and CXCR4, containing the wild-type or mutated binding site of miR-223-3p, were cloned into pmirGLO vectors (Promega, Madison, WI, USA). These plasmids were co-transfected into 293 T cells with NC mimics or miR-223-3p mimics. At 24 h after transfection, the cell culture medium was exhausted, rinsed twice with PBS, added 250 μl of cell lysate. Then the luciferase activity was measured by the luciferase detection kit as per the users’ instructions (KeyGENBioTECH).

### 2.8. Statistical analysis

The quantitative values were expressed as mean ± standard deviation (SD). For more than three groups, statistical significance was assessed by one-way analysis of variance (one-way ANOVA). For the two groups, statistical significance was assessed by the Student’s t-test. *P*< 0.05 was considered statistically significant.

## 3. Results

In our study, we hypothesized that lncRNA DUXAP8 might attenuate PTC via the miR-223-3p/CXCR4 axis. To verify this hypothesis, we conducted the lncRNA DUXAP8-silenced model in two PTC cell lines to investigate the role of DUXAP8 in PTC cell proliferation, migration, invasion and reveal the underlying mechanisms. The effects of miR-223-3p on the malignant phenotype of PTC cells were also evaluated by the up-regulation of miR-223-3p. Further, we also detected the regulatory effect of DUXAP8 on the miR-223-3p/CXCR4 axis.

### 3.1. Knockdown of DUXAP8 suppressed PTC cell proliferation

**Figure 1.**
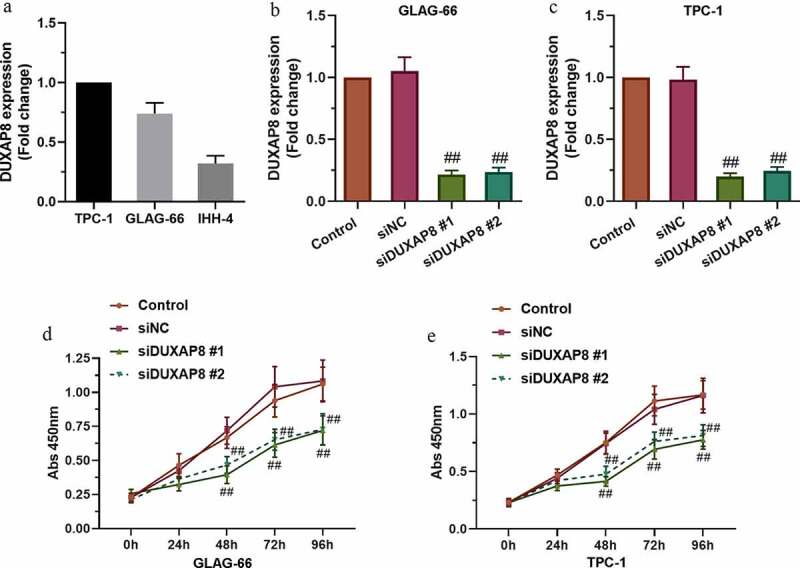
**DUXAP8 expression was upregulated in PTC cell lines and DUXAP8 silencing suppressed PTC cell proliferation**. (a) Expression of DUXAP8 in different PTC cell lines. (b-c) Expression of DUXAP8 in GLAG-66 and TPC-1 cells transfected with DUXAP8 siRNAs. (d-e) The proliferation of GLAG-66 and TPC-1 cells was determined by CCK-8 assay. ## p < 0.01, compared to siNC

GEPIA analysis indicated the positive relationship between high expression of DUXAP8 and the poor prognosis of thyroid cancer (Figure S1). The expression of DUXAP8 in the PTC cell lines, TPC-1, GLAG-66, and IHH-4, was measured by using qRT-PCR assay (Figure 1(a)), then, two cells (TPC-1 and GLAG-66 cells) with higher DUXAP8 expression were selected for subsequent experiments. The cells were transfected with siDUXAP8 or siNC and the mRNA level of DUXAP8 was detected by qRT-PCR. The results showed that DUXAP8 expression was reduced in siDUXAP8#1 and siDUXAP8#2 groups compared with the siNC group (Figure 1(b,c)). Moreover, the CCK-8 assay showed that the knockdown of DUXAP8 suppressed the proliferation of TPC-1 and GLAG-66 cells compared with the siNC group (Figure 1(d,e)). These results demonstrated that DUXAP8 might promote proliferation in PTC cells.

### 3.2. Knockdown of DUXAP8 inhibited migration and invasion of PTC cell lines

**Figure 2.**
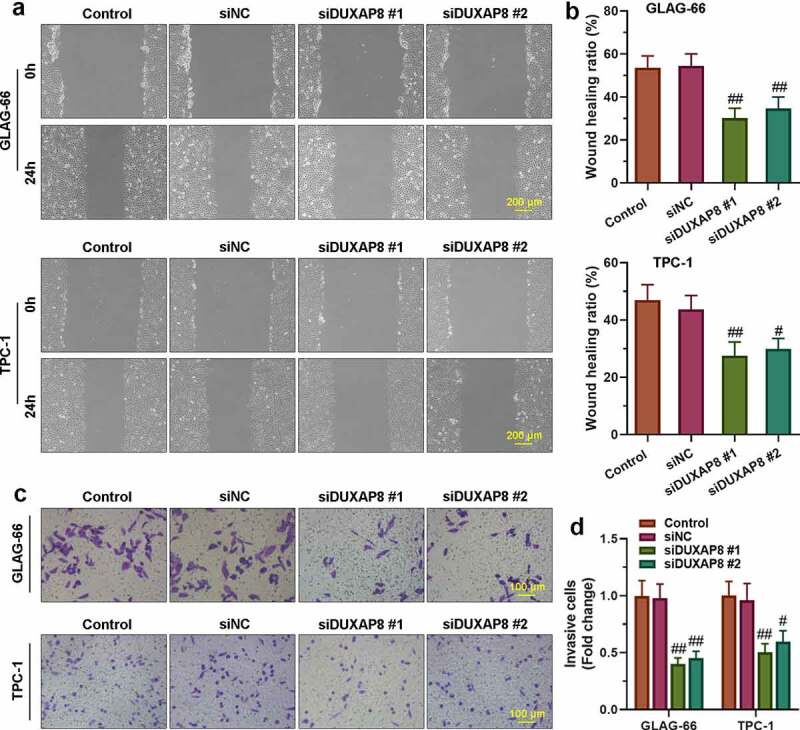
**DUXAP8 silencing inhibited PTC cell migration and invasion**. (a-b) The migration ability of GLAG-66 and TPC-1 cells was assessed using a wound-healing assay. (c-d) The invasive capacity of GLAG-66 and TPC-1 cells was detected through the transwell invasion assay. # p < 0.05, ## p < 0.01, compared to siNC

The cells’ migration invasion abilities were assayed by wound healing and transwell assay. As shown in Figure 2(a,b), the migration rate of TPC-1 and GLAG-66 cells in siDUXAP8#1 and siDUXAP8#2 groups was significantly lower than that in the siNC group, which indicated that knockdown of DUXAP8 inhibited migration of PTC cells. Further, the transwell invasion assay showed that knockdown of DUXAP8 significantly reduced the number of invasive TPC-1 and GLAG-66 cells compared with the siNC group (Figure 2(c,d)), which suggested that down-regulation of DUXAP8 could significantly inhibit the invasion ability of PTC cells.

### 3.3. DUXAP8 bound to miR-223-3p and reduced the expression of miR-223-3p

**Figure 3.**
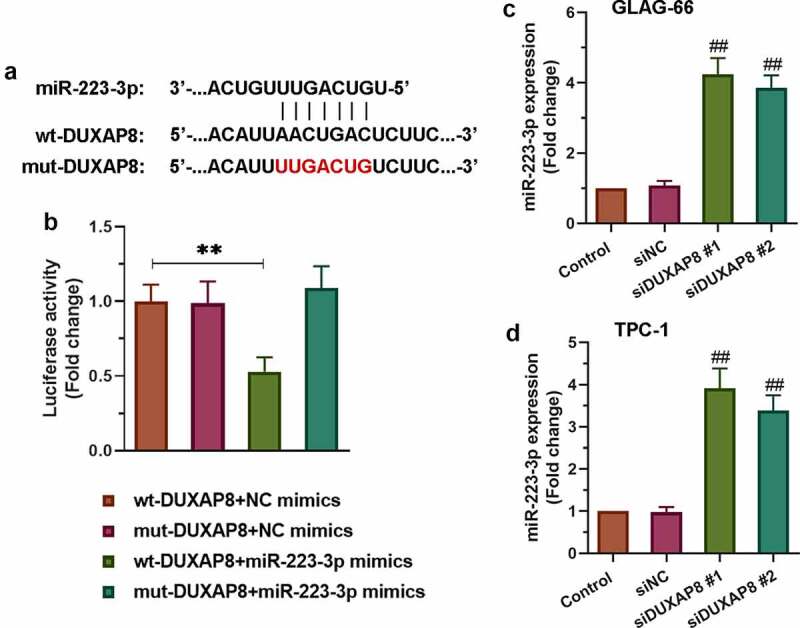
**DUXAP8 bound to miR-223-3p and suppressed the expression of miR-223-3p**. (a) Sequence alignment of DUXAP8 binding to miR-223-3p. (b) The binding activity between DUXAP8 and miR-223-3p was measured by luciferase reporter assay. ** p < 0.01. (c-d) Expression of miR-223-3p in GLAG-66 and TPC-1 cells was examined using qRT-PCR. ## p < 0.01, compared to siNC

To further explore the potential molecular mechanism of DUXAP8 involved in PTC, we searched for ENCORI and found that DUXAP8 had the binding site of miR-223-3p. The sequence alignment of DUXAP8 binding to miR-223-3p was exhibited in Figure 3(a). As showed in Figure 3(b), the luciferase reporter results displayed significantly decreased luciferase activity in the wt-DUXAP8+ miR-223-3p mimics group compared to other groups (p < 0.01). Furthermore, qRT-PCR was used to detect the expression of miR-223-3p after knocking down DUXAP8. The results showed that the expression of miR-223-3p increased significantly after knocking down DUXAP8 in both TPC-1 and GLAG-66 cells (Figure 3(c,d)). Collectively, these data indicated that DUXAP8 acted as a miR-223-3p sponge via the miR-223-3p-binding site.

### 3.4. miR-223-3p over-expression suppressed the proliferation, migration, and invasion of PTC cells

**Figure 4.**
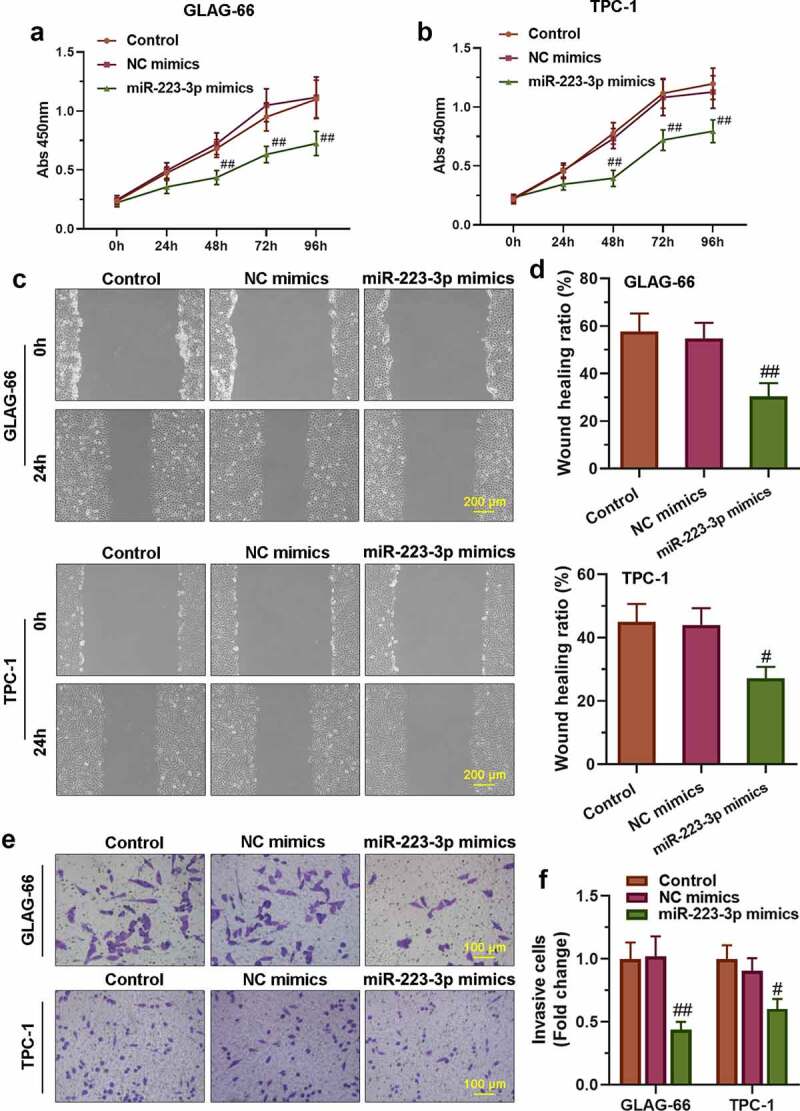
**miR-223-3p over-expression inhibited PTC cell proliferation, migration, and invasion**. (a-b) CCK-8 assay was performed to examine the proliferation of GLAG-66 and TPC-1 cells. (c-d) A wound-healing assay was carried out to measure GLAG-66 and TPC-1 cell migration. (e-f) The transwell invasion assay was used to assess GLAG-66 and TPC-1 cell invasion. # p < 0.05, ## p < 0.01, compared to NC mimics

To further confirm the role of miR-223-3p in regulating the proliferation, migration, and invasion of PTC cells, TPC-1 and GLAG-66 cells were transfected with miR-223-3p mimics or NC mimics. The results of the CCK-8 assay showed that miR-223-3p mimics significantly repressed cell proliferation, as well as migration and invasion activities in TPC-1 and GLAG-66 cells (Figure 4(a–f)). Taken together, these results indicated that over-expression of miR-223-3p inhibited PTC cell proliferation, migration, and invasion.

### 3.5. miR-223-3p targeted 3’UTR of CXCR4 to reduce its expression

**Figure 5.**
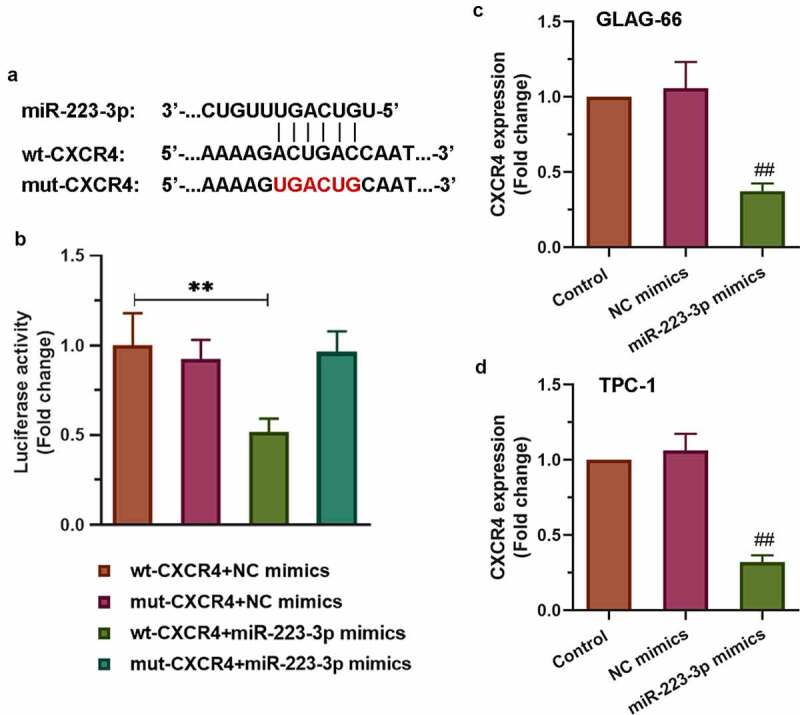
**CXCR4 was a target of miR-223-3p**. (a) Sequence alignment of CXCR4 binding to miR-223-3p. (b) The binding activity between CXCR4 and miR-223-3p was measured by luciferase reporter assay. ** p < 0.01. (c-d) Expression of CXCR4 in GLAG-66 and TPC-1 cells was examined using qRT-PCR. ## p < 0.01, compared to NC mimics

To find the underlying mechanism of miR-223-3p participating in the progression of PTC cell proliferation, migration, and invasion, we searched for ENCORI and found that CXCR4 might be a target gene of miR-223-3p. The sequence alignment of CXCR4 binding to miR-223-3p was exhibited in Figure 5(a). As showed in Figure 5(b), the luciferase reporter results displayed significantly decreased luciferase activity in the wt-CXCR4+ miR-223-3p mimics group compared to other groups (p < 0.01). However, the other two groups (mut-CXCR4+ NC mimics group and mut-CXCR4+ miR-223-3p mimics group) have no effects. Furthermore, qRT-PCR was used to detect the expression of CXCR4. The results showed that over-expression of miR-223-3p reduced CXCR4 expression in both TPC-1 and GLAG-66 cells (Figure 5(c,d)). Collectively, these data indicated that miR-223-3p targeted 3’UTR of CXCR4 to reduce its expression.

### 3.6. DUXAP8 silencing suppressed PTC cell proliferation, migration, and invasion via the miR-223-3p/CXCR4 axis

**Figure 6.**
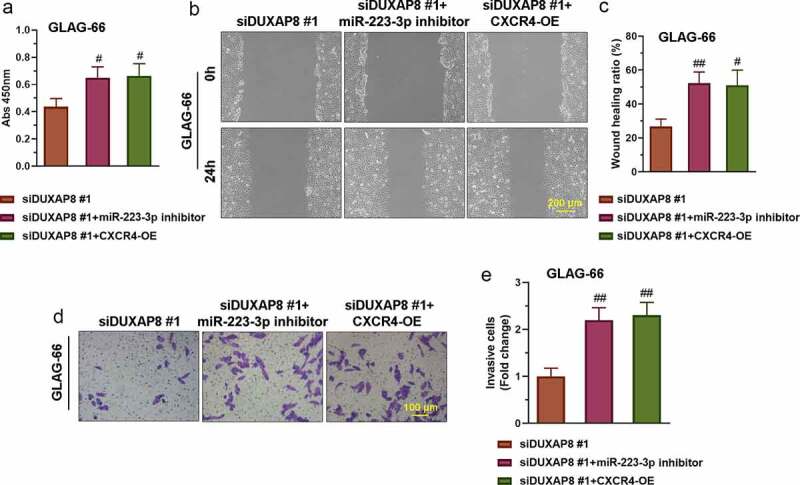
**DUXAP8 silencing suppresses PTC cell proliferation, migration, and invasion via the miR-223-3p/CXCR4 axis**. (a) The proliferation of GLAG-66 cells was tested using CCK-8. (b-c) The migration activity of GLAG-66 cells was detected by the wound healing assay. (d-e) The invasion of GLAG-66 cells was measured by the transwell invasion assay. # p < 0.05, ## p < 0.01, compared to siDUXAP8 #1

The construction of the CXCR4 overexpression (CXCR4-OE) vector refers to the previous articles published by our group [23]. To explore whether DUXAP8 promoted PTC cell proliferation, migration, and invasion through the miR-223-3p/CXCR4 axis, we co-transfect GLAG-66 cells with siDUXAP8#1 and miR-223-3p inhibitor or siDUXAP8#1 and CXCR4-OE respectively. Then CCK-8 assay wound healing assay and transwell invasion assay were respectively used for detecting the proliferation, migration, and invasion of GLAG-66 cells. The results showed that the suppressed proliferation, migration, and invasion activities in DUXAP8 knockdown cells were partly reversed by the inhibition of miR-223-3p or over-expression of CXCR4 (Figure 6(a–e)). These results indicated that DUXAP8 promoted PTC cell proliferation, migration, and invasion through the miR-223-3p/CXCR4 axis.

## 4. Discussion

Worldwide, many registries have reported that the incidence of PTC is arising more and more rapidly [24,25]. Although we have a lot of knowledge about PTC from the perspective of molecular biology, the underlying molecular mechanism of PTC progression is still a mystery.

Recently, there is growing evidence that lncRNAs exert the potential biological functions in regulating different types of cancers [26–29]. Herein, we focus on a pseudogene derived lncRNA DUXAP8. As shown in the previous study, lncRNA DUXAP8 was found to promote gastric cancer (GC) progression and development [16]. Another study sheds light on the critical role of DUXAP8 in regulating the proliferation and invasion of non-small-cell lung cancer cells [15]. However, the biological functional role of DUXAP8 in PTC has not been reported yet. GEPIA analysis results indicated the relationship between DUXAP8 and the prognosis of thyroid cancer (Figure S1). Moreover, our data showed that the knockdown of DUXAP8 inhibited the proliferation, migration, and invasion of PTC cells. Taken together, these results suggested that DUXAP8 might play a pro-tumor role in PTC development.

It is widely known that lncRNA can interact with miRNA as a competitive endogenous RNA (ceRNA), participate in the regulation of target gene expression, and play an important role in the occurrence and development of tumors. To shed light on the mechanism of DUXAP8 in regulating PTC, we searched for ENCORI and found that DUXAP8 might directly target miR-223-3p, which was confirmed by luciferase assay. Several studies have reported the essential role of miR-223-3p in regulating cancers. For example, miR-223-3p repressed the metastasis and progression of osteosarcoma cells by targeting CDH6 [20], over-expression of miR-223-3p suppressed the proliferation, invasion, and migration of breast cancer cells by targeting the ECT2 oncogene [21]. More interestingly, a previous study demonstrated that lower expression of miR-223-3p was found in benign thyroid nodules and PTC in comparison with healthy subjects [30]. Consistent with previous researches, our study demonstrated that the up-regulation of miR-223-3p blocked the malignant phenotype of PTC cells. Further, inhibition of miR-223-3p restored the proliferation, migration, and invasion activities of the DUXAP8 silenced PTC cells, indicating that DUXAP8 may promote the progression of PTC by the regulation of miR-223-3p.

Studies have shown that about 60% of genes have miRNA binding sites in the 3’UTR [31,32]. CXCR4, a chemokine receptor, is out of the common run in regulating various pathological progress, including cancer metastasis, invasion, and migration [33–35]. For instance, a study in breast cancer cells described that promoting effects of Pit-1 in breast cancer metastasis via the CXCL12-CXCR4 axis [36]. Multiple studies indicated that CXCR4 could promote the metastasis, invasion, and migration of gastric cancer [37–39]. Moreover, a previous study had determined the correlation between the expression of CXCR4 and clinicopathological factors in PTC [23]. Besides, He et al. also reported that CXCR4 expression in thyroid lesions was linked with the degree of malignancy [40]. In this study, CXCR4 was proved to be a direct target of miR-223-3p. Over-expression of miR-223-3p reduced the expression of CXCR4 in both TPC-1 and GLAG-66 cells. These results indicate that miR-223-3p can inhibit the proliferation, migration, and invasion of PTC cells by targeting CXCR4. Moreover, DUXAP8 knockdown induced suppression of the malignant behaviors in PTC cells was abrogated by the over-expression of CXCR4, suggesting that DUXAP8 may participate in the malignant progression of PTC through miR-223-3p-mediated regulation of CXCR4.

## 5. Conclusion

In conclusion, it is the first time for our research to reveal that DUXAP8 silencing can suppress the proliferation, migration, and invasion capabilities of PTC, likely via targeting miR-223-3p-mediated regulation of CXCR4. These data validate that DUXAP8 silencing can exert an anti-tumor and anti-metastatic effect and maybe a novel therapeutic target for PTC treatment.

## Supplementary Material

Supplemental Material

## Data Availability

The data used to support the findings of this study are available from the corresponding author upon request. The supplemenary data were aquired from the database (http://gepia.cancer-pku.cn/detail.php?gene=DUXAP8).

## Research highlights

- DUXAP8 silencing suppressed PTC cell proliferation, migration, and invasion
- DUXAP8 bound to miR-223-3p and reduced the expression of miR-223-3p
- The miR-223-3p suppressed the PTC cell proliferation, migration, and invasion
- DUXAP8 promoted PTC progression via the miR-223-3p/CXCR4 axis
