# Supplementary material for: Long non-coding RNA DUXAP8 promotes the cell proliferation, migration, and invasion of papillary thyroid carcinoma via miR-223-3p mediated regulation of CXCR4
Source: Bioengineered. 2021 Feb 15;12(1):496–506. doi: 10.1080/21655979.2021.1882134 (PMC8291844; doi:10.1080/21655979.2021.1882134)
Supplement: Supplemental Material [file KBIE_A_1882134_SM3261.docx]

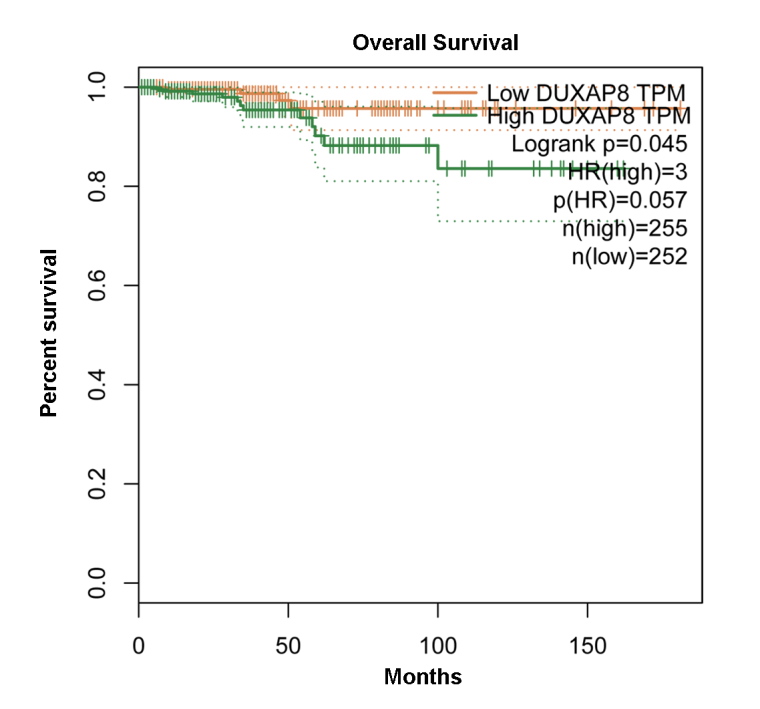


**Supplementary Figure 1**. **The relationship between overall survival and the expression of DUXAP8 in PTC patients from GEPIA database.**
